# Referencing cross-reactivity of detection antibodies for protein array experiments

**DOI:** 10.12688/f1000research.7668.2

**Published:** 2017-05-23

**Authors:** Darragh Lemass, Richard O'Kennedy, Gregor S. Kijanka

**Affiliations:** 1 Biomedical Diagnostics Institute, National Centre for Sensor Research, Dublin City University, Dublin, Ireland; 2 School of Biotechnology, Dublin City University, Dublin, Ireland

**Keywords:** Protein arrays, Whole-cell immunisation, Antibody profiling, Cross-reactivity, Chicken IgY, Reference list, Secondary antibody, Detection antibody

## Abstract

Protein arrays are frequently used to profile antibody repertoires in humans and animals. High-throughput protein array characterisation of complex antibody repertoires necessitates the use of extensively validated secondary detection antibodies. This article details the validation of an affinity-isolated anti-chicken IgY antibody produced in rabbit and a goat anti-rabbit IgG antibody conjugated with alkaline phosphatase using protein arrays consisting of 7,390 distinct human proteins. Probing protein arrays with secondary antibodies in absence of chicken serum revealed non-specific binding to 61 distinct human proteins. Despite the identified non-specific binding, the tested antibodies are well suited for use in protein array experiments as the cross-reactive binding partners can be readily excluded from further analysis. The evident cross-reactivity of the tested secondary detection antibodies points towards the necessity of platform-specific antibody characterisation studies for all secondary immunoreagents. Furthermore, secondary antibody characterisation using protein arrays enables the generation of reference lists of cross-reactive proteins, which can be then marked as potential false positives in follow-up experiments. Providing such cross-reactivity reference lists accessible to the wider research community may help to interpret data generated with the same antibodies in applications not only related to protein arrays such as immunoprecipitation, Western blots or other immunoassays.

## Introduction

Secondary label-conjugated and non-conjugated detection antibodies are frequently used in a wide range of research applications. However, they are often affinity-isolated, polyclonal reagents that may lack the highest standard of antibody validation. The antibodies characterised in this study are a polyclonal anti-chicken IgY antibody produced in rabbit (31104, Thermo Fisher) and a polyclonal goat anti-rabbit IgG antibody conjugated with alkaline phosphatase (AP) (A3687, Sigma-Aldrich). Although the use of the rabbit anti-IgY antibody in the literature is limited, the goat anti-rabbit IgG AP has been extensively utilised for over 15 years ^1, 2^.

The research conducted in this laboratory examines complex antibody repertoires in humans and animals by means of protein arrays. Protein arrays are frequently used to profile antibody binding to human proteins in autoimmune disease ^3^, cancer ^4^ and in healthy individuals ^5^. Other protein array applications include recombinant ^6^ and hybridoma-derived ^7^ antibody characterisation studies. This article investigates the cross-reactivity of a rabbit anti-chicken IgY and an alkaline phosphatase-conjugated goat anti-rabbit IgG, which were used for the profiling of IgY antibody responses to human antigens in chickens immunised with human cancer cells. The protein array technology applied here, developed by Büssow and colleagues ^8^, is comprised in its current version of a fully annotated set of 7,390 distinct human proteins, that may serve as potential antigens. The aim of this study is to define a cross-reactivity reference list for the two described secondary antibodies, which can then be used to eliminate non-specific binders from ongoing chicken IgY profiling studies. Furthermore, publication of the cross-reactivity reference list provides a valuable resource of potential false-positive binders to researchers using the same antibodies. may support other researchers using these antibodies in the evaluation of their experiments.

## Materials and methods

### Antibody details

Rabbit anti-chicken IgY (H+L) secondary antibody (Thermo Fisher Scientific, Product number 31104, Lot code PK19380211) is a polyclonal antibody that targets the variable heavy and light chains of chicken IgY immunoglobulins ( Table 1). The antibody was isolated from the serum of the antigen-immunised rabbit through immunoaffinity chromatography using antigen coupled to agarose beads. The antibody was added to the protein array at a 1/1,000 dilution in 2% (w/v) bovine serum albumin (BSA, Sigma-Aldrich, A2153) in tris-buffered saline (TBS, Trizma ^®^ Base, Sigma-Aldrich, T6066 and sodium chloride, Fisher Scientific, S/3160/68) with 0.1%, v/v, Tween 20 (Sigma-Aldrich, P1379).

**Table 1.** Details of characterised antibodies.

Antibody	Manufacturer	Catalogue Number	Lot Number	Stock Concentration	RRID
Rabbit anti-Chicken IgY (H+L)	Thermo Fisher Scientific	31104	PK19380211	2.3 mg/mL	AB_228382
Goat anti-rabbit IgG Alkaline Phosphatase conjugated	Sigma-Aldrich	A3687	SLBJ6146V	4.0 mg/mL	AB_258103

Alkaline phosphatase-conjugated goat anti-rabbit IgG (whole molecule) (Sigma-Aldrich, Product number A3687, Lot code SLBJ6146V) is a polyclonal antibody that targets all rabbit IgGs ( Table 1). The antibody was isolated through immunospecific purification of antisera from a rabbit IgG-immunised goat. Following isolation, the anti-rabbit IgG was conjugated to alkaline phosphatase using glutaraldehyde-based cross-linkage. The antibody was added to the protein array at a 1/1,000 dilution in 2% (w/v) BSA in tris-buffered saline (TBS) with 0.1%, v/v, Tween 20.

### Protein arrays

Unipex protein arrays were obtained from Source Bioscience Life Sciences (Nottingham, UK). The Unipex arrays comprise of 15,300 fully annotated *E. coli* clones expressing a total of 7,390 distinct in-frame ORF human recombinant proteins. The Unipex proteins are immobilized under denaturing conditions directly on the PVDF membrane surfaces exposing linear sequence epitopes ideally suited for epitope mapping, antibody profiling and antibody cross-reactivity analyses. The details of protein arrays utilised in this study are provided in Table 2. For general information on Unipex protein arrays please refer to: ( http://www.lifesciences.sourcebioscience.com/media/290406/sbs_ig_manual_proteinarray_v1.pdf).

**Table 2.** Details of protein arrays.

Protein array	Library Number	Array Number	Manufacturer
Unipex 1 pt.1	9027	633.4.730	Source Bioscience
Unipex 2 pt.1	9028	634.5.737	Source Bioscience

### Cross-reactivity assessment

**Figure 1.**
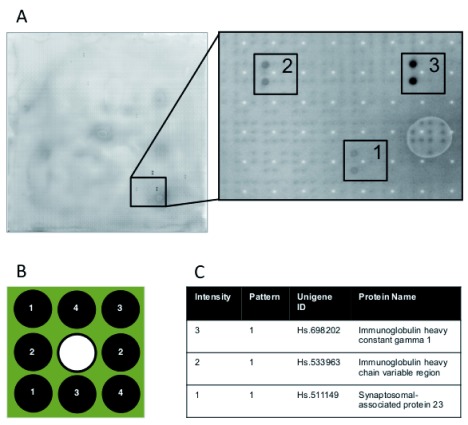
Cross-reactivity of rabbit anti-chicken IgY and goat anti-rabbit IgG identified by protein array screening. ( **A**) Image of a whole protein array and a representative section illustrating antibody-antigen binding at three different signal intensities; 3 = strong, 2 = intermediate and 1 = weak. ( **B**) The proteins are arranged in a 3×3 pattern on the array and all proteins are arrayed twice and appear as duplicate spots in a particular pattern within a block after a successful hybridization. ( **C**) Description of proteins chosen as examples provided on the representative array image above; signal intensities, patterns, Unigene IDs and protein names are listed.

Antibody cross-reactivity was assessed using Unipex protein arrays. The detailed experimental protocol is provided in Table 3. Briefly, secondary rabbit anti-chicken IgY and goat anti-rabbit IgG AP were validated in preparation for a chicken IgY antibody profiling experiment of a chicken immunised with human cancer cells. Protein arrays were probed with secondary antibodies in the absence of IgY-containing chicken serum, as described in Table 3. Signal generation for array-bound secondary antibodies was obtained using AttoPhos AP fluorescent substrate system (Promega, S1001) diluted 1 in 8 in AP buffer (1mM MgCl2, Sigma-Aldrich, M4880 and 100mM Tris base, pH 9.5). Protein array image acquisition was conducted using a Fuji scanner Fla5100. Positive signals were localized according to the manufacturer’s protocol. Briefly, array proteins were spotted in duplicate in a 3×3 square pattern. The centre spot of each square being a guide dot surrounded by eight flanking protein spots. Each protein was spotted around the navigation dot in one of four predetermined patterns (see Figure 1b). Varying background intensities were controlled by adjusting brightness and contrast of the image using Visual Grid software (GPC Biotech) to allow best possible scoring conditions. The degree of signal intensity was evaluated for each protein pair with the value 1 corresponding to a weak signal, value 2 corresponding to a moderate signal and value 3 corresponding to a strong signal. The x- y- coordinates of each positive pattern were merged with the Unipex protein database provided by the manufacturer (Source Bioscience) resulting in identification of GenBank and UniGene ID’s for each positive signal. This is a commonly used method for scoring signal intensities as previously shown by this group ^4^ and others ^9, 10^. Protein annotations were retrieved from the Unipex database provided by the manufacturer and updated using the National Cancer Institute’s UniGene CGAP Gene Finder tool ( http://cgap.nci.nih.gov/Genes/GeneFinder).

**Table 3.** Secondary antibody protein array analysis protocol.

Protocol steps	Objective	Reagent	Time
Protein array preparation	Rinse array	70% (v/v) ethanol	5 min
	Remove ethanol and rinse	dH _2_0 for 2	2 min
	Wipe off all *E. coli* colonies	laminar tissue	As appropriate
Wash 1	Wash off any *E. coli* debris	TBST-T	10min (x3)
		TBS	2min (x2)
		TBS	10 min
Array blocking	Block arrays by shaking	5% (w/v) Milk Marvel TBS-T	2h
Wash 2	Wash off any Blocking solution	TBS-T	15min (x3)
Incubate first antibody	Rabbit anti-chicken IgY	1 in 1,000 (no recommended western dil.) in 2% (w/v) BSA TBS-T	2h
Wash 2	Wash off any unbound antibody	TBS-T	15 min (x3)
Incubate second antibody	Goat anti-rabbit IgG-AP	1 in 1,000 in 2% (w/v) BSA TBS-T	2h
Wash 3	Wash off any unbound antibody	TBS-T	10 min (x2)
		TBS	10 min (x2)
Protein array signal detection	Signal generation for array bound goat anti-rabbit IgG-AP	AttoPhos AP Fluorescent Substrate diluted 1 in 8 in AP buffer (1mM MgCl _2_, 100mM Tris base, pH 9.5)	10 min
	Protein array image acquisition	FujiScanner Fla5100 (Settings Laser: 473, Filter: LPB, Resolution 50μm	18 min

### Epitope analysis

To investigate whether antibody binding to protein arrays was due to epitope similarities between the animal immunogens used to produce the secondary antibodies and the human proteins on the arrays we performed a comparative analysis as follows. Sequences of human antigens on the array bound by the secondary antibodies were obtained from the PubMed website ( http://www.ncbi.nlm.nih.gov/protein/) using IDs present in the Unipex protein database and compared to chicken immunoglobulin proteins [Ig lambda chain C region (NCBI Accession: P20763.1), Ig lambda chain V-1 region (NCBI Accession: P04210.1), immunoglobulin Y heavy chain constant region (NCBI Accession: XP_015130394.1) and immunoglobulin Y heavy chain variable region (NCBI Accession: ADF29959.1)], as well as to rabbit immunoglobulin proteins [Ig gamma chain C region (UniProtKB: P01870), immunoglobulin heavy chain VDJ region, partial (NCBI Accession: AAA51320.1), Ig lambda chain C region (UniProtKB: P01847.2) and Ig lambda chain variable region, partial (NCBI Accession: AAA31364.1)]. For antigen similarity comparisons, sequence similarities were analysed using BLAST. Non-intersecting protein sequence alignments were analysed using the local similarity program SIM adjusted to the BLOSUM62 comparison matrix to ensure amino acid complementarity of linear B-cell epitopes as previously shown ^11^. The threshold for sequence similarity was set to BLAST E-values below 1 × 10 ^-10^ and SIM score values above 50.

## Results

Probing protein arrays with antibodies allows the assessment of their specificity and cross-reactivity across a large numbers of potential antigens in parallel ^12, 13^. Here we investigated the cross-reactivity of a secondary rabbit anti-chicken IgY and a goat anti-rabbit IgG labelled with AP, using a single set of human protein arrays in the absence of chicken serum. We identified a total of 63 binding events, of which 61 corresponded to unique proteins ( Table 4). The identified positive signals varied in strength, as shown in Figure 1, with intensity 3 being the strongest and 1 the weakest. Five of the identified signals were scored as intensity 3, twelve signals were scored as intensity 2 and remainder scored as intensity 1. The original protein array images are shown in Figure S1 and Figure S2 ( Supplementary material) and protein array images with highlighted positive signals, which correspond the cross-reactive proteins listed in Table 4, are shown in Figure S3 and Figure S4 ( Supplementary material).

**Table 4.** Reference list of antibody cross-reactivity identified by protein array analysis.

Protein array clone ID	Signal Intensity ^*^	GenBankID	UnigeneID	Name
IMGSp9028F0610D	3	BM914329	Hs.533963	Clone SFV019_2F05H immunoglobulin heavy chain variable region
IMGSp9028H079D	3	BQ711793	Hs.547404	Clone IgA-MZ-aa42c-2 immunoglobulin alpha heavy chain variable region (IgA)
IMGSp9028F0316D	3	BQ709082	Hs.620437	IGH mRNA for immunoglobulin heavy chain VHDJ region, partial cds, clone:TRH1-16
IMGSp9028G0921D	3	BX417981	Hs.698070	Immunoglobulin heavy constant gamma 1 (G1m marker)
IMGSp9027F0514D	3	118471	Hs.15951	Proline-rich acidic protein 1
IMGSp9027H0434D	2	BC044933	Hs.135094	Kinesin family member 18B
IMGSp9027G0658D	2	BC010132	Hs.445893	KH domain containing, RNA binding, signal transduction- associated 1
IMGSp9027F0369D	2	AK092483	Hs.470417	Penta-EF-hand domain containing 1
IMGSp9027D1015D	2	NM_006814	Hs.471917	Proteasome (prosome, macropain) inhibitor subunit 1 (PI31)
IMGSp9028C0313D	2	DA970556	Hs.510650	Clone IP80 immunoglobulin heavy chain variable region
IMGSp9027G0525D	2	NM_002228	Hs.525704	Jun proto-oncogene
IMGSp9027E0966D	2	BC041022	Hs.584909	SCAN domain containing 1
IMGSp9027F0171D	2	BC018708	Hs.632706	Zinc finger CCCH-type containing 10
IMGSp9027F0625D	2	BC018708	Hs.632706	Zinc finger CCCH-type containing 10
IMGSp9028G0311D	2	BM920476	Hs.633485	Enhancer of polycomb homolog 1 (Drosophila)e
IMGSp9028G027D	2	BX417981	Hs.698070	Immunoglobulin heavy constant gamma 1 (G1m marker)
IMGSp9028F099D	2	BG754662	Hs.698202	UniGene entry Hs.698202 has been retired; current entry: Transcribed locus, moderately similar to XP_001496515.2 PREDICTED: ig gamma-3 chain C region [Equus caballus]
IMGSp9027H0728D	1	NM_001978	Hs.106124	Erythrocyte membrane protein band 4.9 (dematin)
IMGSp9027C0116D	1	NM_080881	Hs.130316	Drebrin 1
IMGSp9027D129D	1	NM_001012426	Hs.131436	Homo sapiens forkhead box P4 (FOXP4), transcript variant 1, mRNA
IMGSp9027G0310D	1	BX647115	Hs.173381	Dihydropyrimidinase-like 2
IMGSp9027G0172D	1	AF479827	Hs.182081	BR serine/threonine kinase 1
IMGSp9028E0623D	1	NM_022489	Hs.24956	Inverted formin, FH2 and WH2 domain containing
IMGSp9027C0164D	1	BC000786	Hs.25584	ADP-ribosylation factor GTPase activating protein 1
IMGSp9027A0339D	1	BC008343	Hs.292493	X-ray repair complementing defective repair in Chinese hamster cells 6
IMGSp9027C1211D	1	BC000459	Hs.306791	Polymerase (DNA directed), delta 2, accessory subunit
IMGSp9027E0916D	1	BC040880	Hs.315568	Chromosome 10 open reading frame 114
IMGSp9027H0366D	1	NM_003260	Hs.332173	Transducin-like enhancer of split 2 (E(sp1) homolog, Drosophila)
IMGSp9028A0867D	1	AL833379	Hs.333388	Eukaryotic translation elongation factor 1 delta (guanine nucleotide exchange protein)
IMGSp9027F1049D	1	NM_006548	Hs.35354	Insulin-like growth factor 2 mRNA binding protein 2
IMGSp9027G1059D	1	AK097073	Hs.361323	ATP-binding cassette, sub-family F (GCN20), member 3
IMGSp9027H0825D	1	AK127401	Hs.407368	LSM14A, SCD6 homolog A (S. cerevisiae)
IMGSp9028A0819D	1	BC036307	Hs.465929	Calponin 1, basic, smooth muscle
IMGSp9027D1063D	1	BC146654	Hs.493796	RUN and SH3 domain containing 2
IMGSp9028D0712D	1	BC022890	Hs.511149	Synaptosomal-associated protein, 23kDa
IMGSp9027F0118D	1	NM_002087	Hs.514220	Granulin
IMGSp9027B0725D	1	NM_032627	Hs.515259	Single stranded DNA binding protein 4
IMGSp9027G1020D	1	AK127255	Hs.515364	Rho GTPase activating protein 33
IMGSp9027F0926D	1	BC090883	Hs.516160	Splicing factor 3b, subunit 4, 49kDa
IMGSp9027H0318D	1	NM_003768	Hs.517216	Phosphoprotein enriched in astrocytes 15
IMGSp9027B0425D	1	AB002328	Hs.517478	Calcineurin binding protein 1
IMGSp9027D0732D	1	AK096320	Hs.517543	Pescadillo ribosomal biogenesis factor 1
IMGSp9027E0219D	1	NM_015695	Hs.520096	Bromodomain and PHD finger containing, 3
IMGSp9027B0369D	1	NM_007371	Hs.522472	Bromodomain containing 3
IMGSp9027H1010D	1	NM_014866	Hs.522500	SEC16 homolog A (S. cerevisiae)
IMGSp9027B0757D	1	NM_031372	Hs.527105	Heterogeneous nuclear ribonucleoprotein D-like
IMGSp9027C0965D	1	NM_014811	Hs.533260	Protein phosphatase 1, regulatory subunit 26
IMGSp9027E0964D	1	NM_001098800	Hs.571729	Melanoma antigen family D, 4
IMGSp9027G0156D	1	BC037307	Hs.590990	Anoctamin 8
IMGSp9027C1216D	1	AB208876	Hs.592082	Axin 1
IMGSp9027F0322D	1	BF110897	Hs.612694	Transcribed locus
IMGSp9027E0122D	1	BC004352	Hs.613351	Kinesin family member 22
IMGSp9027G0312D	1	AK225632	Hs.631593	Protein phosphatase 1, regulatory subunit 15A
IMGSp9027E104D	1	AL133055	Hs.636446	Zinc finger protein 853
IMGSp9027A1171D	1	NM_032329	Hs.645460	Inhibitor of growth family, member 5
IMGSp9027B0415D	1	XR_015693	Hs.654404	Major histocompatibility complex, class I, B
IMGSp9027A0861D	1	BC006105	Hs.654798	Alpha tubulin acetyltransferase 1
IMGSp9027F0471D	1	NM_002140	Hs.695973	Heterogeneous nuclear ribonucleoprotein K
IMGSp9027D0731D	1	NM_001270	Hs.696018	Chromodomain helicase DNA binding protein 1
IMGSp9027B0439D	1	AK124880	Hs.696054	Protein phosphatase 1, regulatory subunit 18
IMGSp9027C0140D	1	AB209272	Hs.76662	Zinc finger, DHHC-type containing 16
IMGSp9027C1264D	1	CR606311	Hs.77100	General transcription factor IIE, polypeptide 2, beta 34kDa
IMGSp9027E1075D	1	AK128584	Hs.79110	Nucleolin

The 61 identified proteins comprised of a wide range of human proteins, including immunoglobulins, as well as a variety of nuclear, cytoplasmic and cell-membrane proteins with a diverse range of functions ( Table 4). In order to identify shared epitopes that could explain the observed antibody cross-reactivity, and to deduce the origin of the non-specific binding to either of the two tested antibodies, we investigated sequence similarities between the human proteins and the immunogens used to produce the antibodies. We conducted a linear (BLAST) and a segmented (SIM) *in silico* sequence analysis of chicken IgY and rabbit IgG immunoglobulins against 61 array-identified human proteins as detailed in supplementary Table 1. In total, 5 proteins met the BLAST threshold criteria of E-values below 1 × 10 ^-10^, as well as the SIM threshold criteria of scores above 50. A further 9 proteins met the SIM threshold, but they did not meet the BLAST criteria ( Table 5).

All 5 proteins that met both threshold criteria belong to the immunoglobulin class of proteins, four being variable regions of Ig heavy chains and one a heavy constant gamma chain. The *in silico* sequence analysis revealed the highest sequence similarity to Ig heavy variable and constant chains, respectively, of both, the chicken IgY and rabbit IgG in all cases ( Supplementary Table 1) making it impossible with this approach to deduce the origin of the cross-reactivity.

**Table 5.** *In silico* sequence similarity analysis between chicken IgY and rabbit IgG and array-identified human proteins. 14 most similar human proteins detected in this study, prioritised by sequence similarity.

Human protein	Chicken Immunoglobulins				Rabbit Immunoglobulins				Signal Intensity
	BLAST overlaps ^*^	Highest BLAST E-value	SIM overlaps ^*^	Highest Score	BLAST overlaps ^*^	BLAST	SIM overlaps ^*^	Highest Score	
Clone SFV019_2F0 5H immunoglobulin heavy chain variable region	1	1.00E-48	2	393	2	3.00E-47	4	356	3
Clone IgA-MZ-aa42c-2 immunoglobulin alpha heavy chain variable region (IgA)	1	4.00E-30	2	210	1	1.00E-29	1	194	3
IGH mRNA for immunoglobulin heavy chain VHDJ region, partial cds, clone:TRH1-16	2	2.00E-34	1	218	1	1.00E-41	2	291	3
Immunoglobulin heavy constant gamma 1 (G1m marker)	1	3.00E-13	1	86	2	2.00E-125	2	890	3
Clone IP80 immunoglobulin heavy chain variable region	1	3.00E-44	1	313	1	3.00E-49	2	357	2
Enhancer of polycomb homolog 1 (Drosophila)e	0	BT	1	51	0	BT	0	BT	2
Inverted formin, FH2 and WH2 domain containing	0	BT	0	NA	0	BT	1	51	1
Chromosome 10 open reading frame 114	0	BT	1	51	0	BT	0	BT	1
RUN and SH3 domain containing 2	0	BT	1	52	0	BT	0	BT	1
Single stranded DNA binding protein 4	0	BT	1	56	0	BT	0	BT	1
Rho GTPase activating protein 33	0	BT	1	54	0	BT	0	BT	1
Splicing factor 3b, subunit 4, 49kDa	0	BT	1	61	0	BT	0	BT	1
Protein phosphatase 1, regulatory subunit 26	0	BT	1	59	0	BT	0	BT	1
Alpha tubulin acetyltransferase 1	0	BT	1	50	0	BT	0	BT	1

‘BT’ signifies ‘Below Threshold Value’. Threshold values were BLAST E-values below 1 × 10 ^-10^ and SIM values above 50. ‘NA’ signifies ‘No significant similarity was detected’ ^*^BLAST and SIM overlaps indicate the number of sequence categories meeting threshold criteria for similarity as shown in Supplementary Table 1.

The 9 proteins that met only the SIM threshold criteria belong to a wide range of protein classes, however, none of those proteins belongs to the immunoglobulin class of proteins. In silico sequence analysis revealed that 8 of those proteins have a high local sequence similarity to the chicken immunoglobulin Y heavy chain constant region, but not to any other chicken and rabbit Ig regions. The analysis revealed further, that Inverted Formin, FH2 and WH2 Domain Containing (INF2) showed high local similarity exclusively to the rabbit Ig gamma chain constant region ( Supplementary Table 1).

## Conclusion

This work illustrates the cross-reactivity of an antibody-based detection system for IgY binding. The polyclonal anti-IgY rabbit antibody in combination with an anti-rabbit IgG alkaline phosphatase-conjugated antibody was shown to bind to 61 human proteins present on Unipex protein arrays comprising of 7,390 human proteins. Characterisation of this cross-reactivity provides a ‘false-positive’ database for future chicken antisera characterisation on protein array systems not limited to the Unipex protein array used here. These results, in combination with ‘false-positives’ from earlier research investigating antibody cross-reactivity by this group ^12^ and others ^13^ may provide valuable information for future protein array-based experiments. Reference lists provided by such experiments would be further strengthened by arrays that include additional portions of the human proteome and/or post-translational modifications. Using antibodies that have been extensively characterised on protein arrays will reduce the risk of identifying irrelevant cross-reactive secondary antibody binding to the array as a host-antigen response.

It is important to note that the current study was a one-off experiment and repeat experiments may increase the reliability of the data. The reproducibility of the binding events identified in this study was further warranted by evaluating each protein in two discrete positions on the array. Of the 63 binding events, five were scored as intensity 3, twelve were scored as intensity 2 and the remainder were intensity 1. While the assay is unable to conclusively distinguish the precise cause of the differences in signal intensities, it can be assumed to be due to variations in antibody affinity and avidity, the availability of the epitope for binding, and protein concentrations on the array. A follow-up quantitative Western blot analyses and titration experiments would help further to shed more light into differences in antigen-binding kinetics.

The secondary antibodies utilized in this study are polyclonal, isolated by immunoaffinity chromatography. The presented cross-reactivity reference list may, therefore, show some variation when a different lot of the antibody is used. We have previously shown that conditions applied during affinity chromatography may affect specificity ^14^. When assessing protein array images, we found a considerable discrepancy in background intensity of array part 1 and 2. It is important to highlight that the part 1 and part 2 of the array are generated from distinct clone libraries of different tissue origin. Part 1 of the array was generated from human brain tissue using a pQE30NST vector, whereas part 2 of the array was generated from different sources of tissue, including T cells and lung tissue, using a pQE80LSN vector. The tissue origin and the utilised bacterial vector are potential contributing factors for the variances in background noise.

Since both antibodies were used as a pair in this study, it was not possible to directly deduce the exact cross-reactivity profile for each individual antibody. We have therefore taken an *in silico* sequence analysis approach and we found that five of the identified proteins were of the immunoglobulin class of proteins with very high sequence similarities to both, the chicken IgY and the rabbit IgG immunoglobulins. Such cross-reactivity is not surprising considering that the antibodies are polyclonal and the immunogens were immunoglobulins of both hosts. In addition, the data sheet provided with the anti-chicken IgY antibody produced in rabbit (31104, Thermo Fisher) has specified that this antibody may cross-react with immunoglobulins from other species. The data sheet for the goat anti-rabbit IgG AP antibody (A3687, Sigma-Aldrich) has specified binding to all rabbit immunoglobulins. The *in silico* sequence analysis revealed furthermore 8 proteins with high sequence similarity to chicken IgY heavy chain constant region and one protein with high sequence similarity to rabbit Ig gamma chain constant region. In order to tackle this issue experimentally, a single labelled antibody should be tested on its own in future experiments. Furthermore, if a non-labelled antibody is to be tested, two experiments should be performed, one with a labelled and non-labelled antibody pair such as demonstrated in this study, and one additional experiment with the labelled antibody alone, thereby allowing allocation of exact cross-reactivates by simply subtracting ‘false-positives’ from both sets of results.

In conclusion, the antibodies tested in this study showed cross-reactivity to unrelated human proteins as well as to human immunoglobulin proteins, which are homologous to the original immunogens. Despite the identified non-specific binding, the tested antibodies are suitable for use in protein array experiments as the cross-reactive binding partners can be readily excluded from further analysis. As both antibodies were used as a pair in this study, the possibility to deduce the exact cross-reactivity profile for each individual antibody may be limited. However, the cross-reactivity reference list provided in this paper can be further utilised to validate research using those antibodies in applications other than protein arrays.

## Supplementary material

In order to investigate whether potential shared epitopes exist between immunogens (chicken IgY, rabbit IgG) used to produce the secondary antibodies tested in this study and the human proteins bound non-specifically by those secondary antibodies on protein arrays, we analysed sequence similarities using BLASTP and the local similarity program SIM. We used sequences of protein IDs provided by the Unipex protein database and compared those to chicken and rabbit immunoglobulin proteins (Methods section, Epitope analysis). The Table S1 shows the results for all possible two-pair sequence alignment combinations presented as BLAST scores, E-values and SIM scores.

Original image of protein array (Number 633.4.730) probed with rabbit anti-chicken IgY and alkaline phosphatase-conjugated goat anti-rabbit IgG, visualised using AttoPhos AP Fluorescent Substrate.

Original image of protein array (Number 634.5.737) probed with rabbit anti-chicken IgY and alkaline phosphatase-conjugated goat anti-rabbit IgG, visualised using AttoPhos AP Fluorescent Substrate.

Cross-reactive proteins listed in Table 4 are highlighted corresponding to their intensity as red (intensity 3 = strong), green (intensity 2 = intermediate) and yellow (intensity 1 = weak) circles.

Cross-reactive proteins listed in Table 4 are highlighted corresponding to their intensity as red (intensity 3 = strong), green (intensity 2 = intermediate) and yellow (intensity 1 = weak) circles.
