# Supplementary figures and images for: Referencing cross-reactivity of detection antibodies for protein array experiments
Source: F1000Res. 2017 May 23;5:73. Originally published 2016 Jan 18. [Version 2] doi: 10.12688/f1000research.7668.2 (PMC4893991; doi:10.12688/f1000research.7668.2)

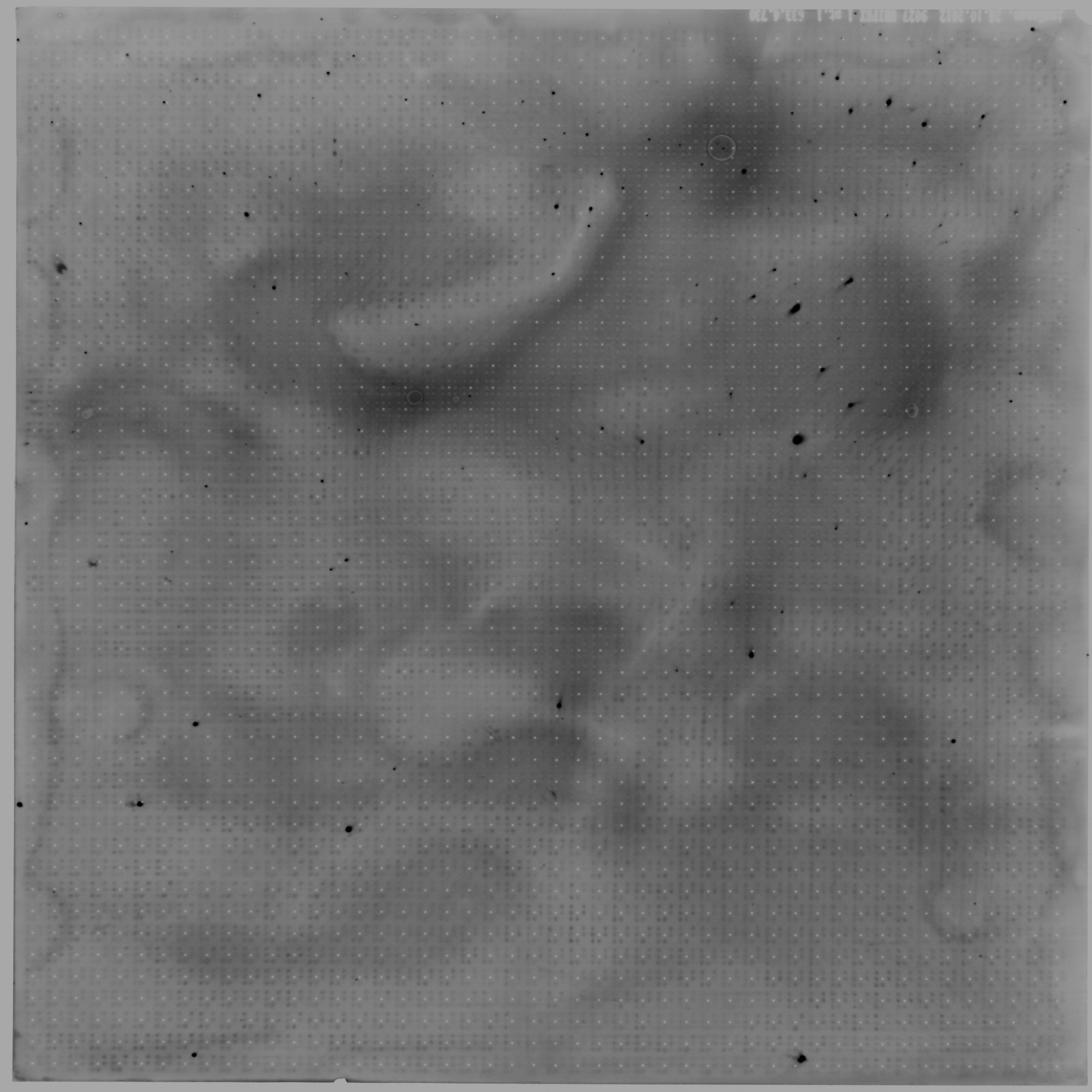

Supplement: Supplementary file 2 [file f1000research-5-12612-s0000.tgz › 3f6c2af3-086b-4ad2-b92b-b1e6005b917a.tif]

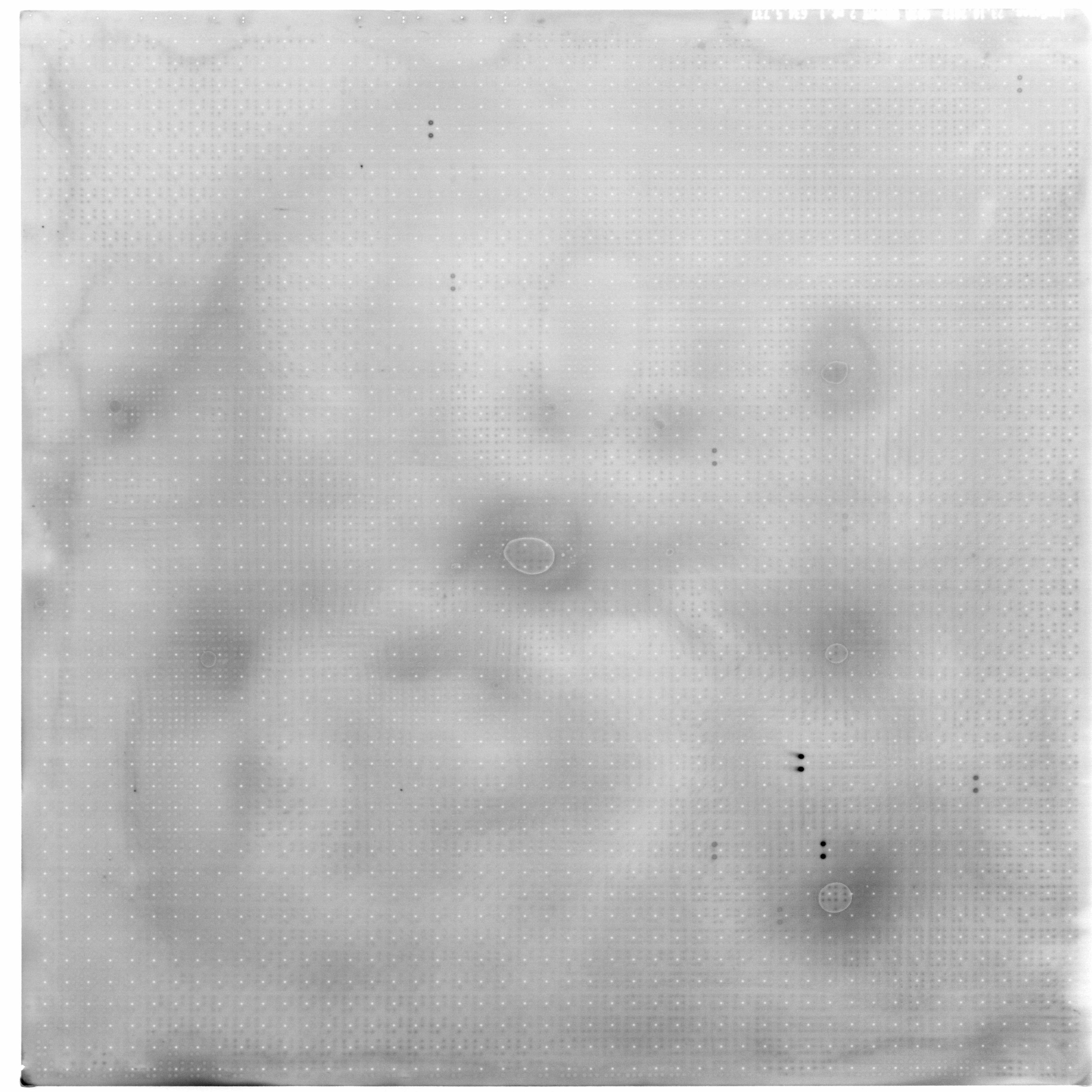

Supplement: Supplementary file 3 [file f1000research-5-12612-s0001.tgz › dc3a239b-1eba-4591-b78d-122441a456b6.tif]

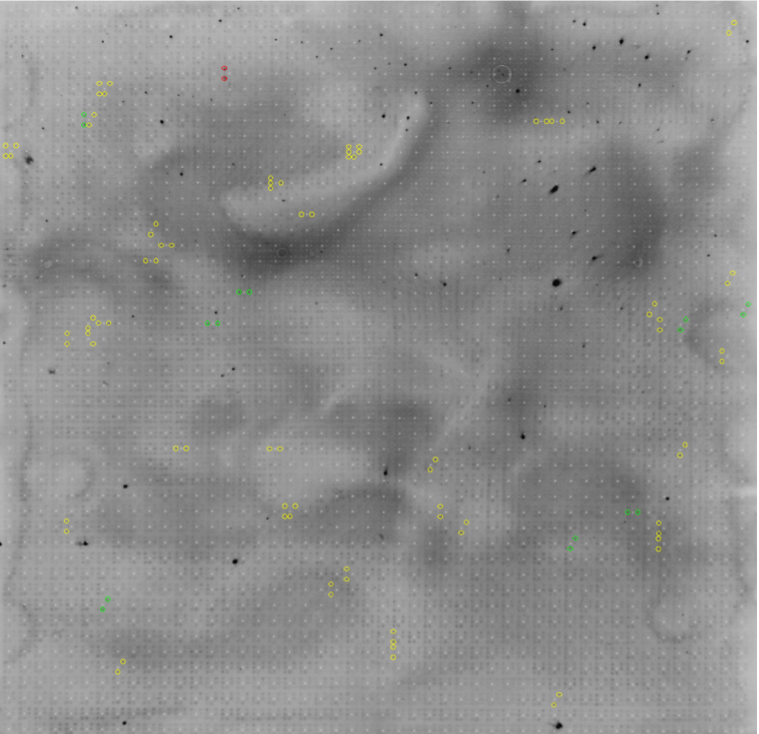

Supplement: Supplementary file 4 [file f1000research-5-12612-s0002.tgz › f0f4843b-0901-4554-a275-362f15fad673.tiff]

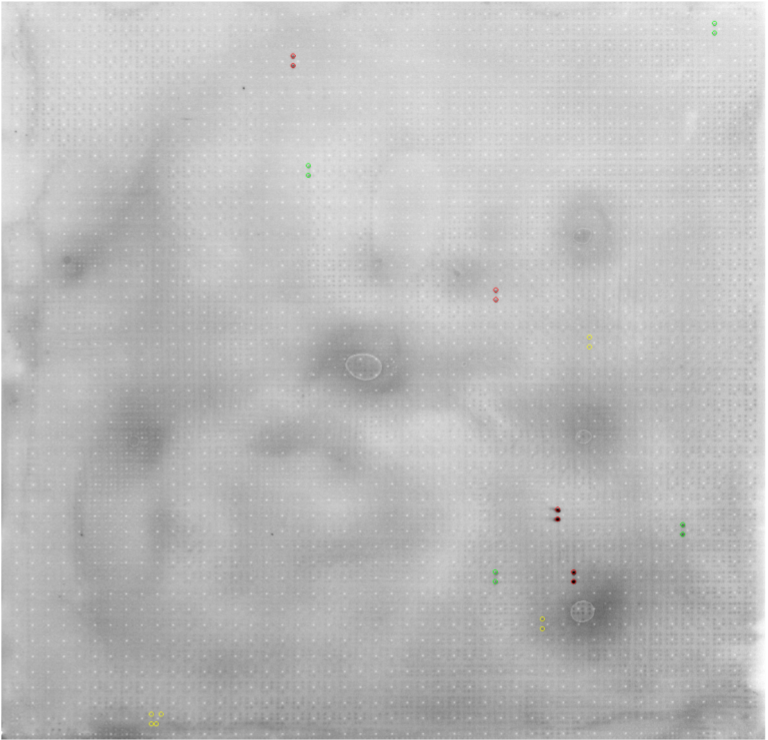

Supplement: Supplementary file 5 [file f1000research-5-12612-s0003.tgz › dd99a1dc-5833-43a1-a28f-95cc9a378172.tiff]
