# Supplementary material for: Referencing cross-reactivity of detection antibodies for protein array experiments
Source: F1000Res. 2017 May 23;5:73. Originally published 2016 Jan 18. [Version 2] doi: 10.12688/f1000research.7668.2 (PMC4893991; doi:10.12688/f1000research.7668.2)
Supplement: Peer review history

# Peer review: Referee response for version 2

**DOI:** 10.5256/f1000research.12612.r22973

**Published:** 2017-06-02

**Authors:** Konrad Büssow

**Affiliations:** 1 Structural Biology, Helmholtz Center for Infection Research, Braunschweig, Germany

The authors have improved their manuscript considerably. The last sentence of the Introduction needs correction. Otherwise, the paper has been sufficiently improved for publication.

I have read this submission. I believe that I have an appropriate level of expertise to confirm that it is of an acceptable scientific standard.

# Peer review: Referee response for version 1

**DOI:** 10.5256/f1000research.8257.r12773

**Published:** 2016-04-04

**Authors:** Konrad Büssow

**Affiliations:** 1 Structural Biology, Helmholtz Center for Infection Research, Braunschweig, Germany

In the present work, the authors have tested direct binding of secondary antibodies to arrays of human proteins.

Readers who use array technology may benefit from the present work, since they will become aware of the problem of signals caused by secondary antibodies and not by the primary antibody. It appears that human immunoglobulins are frequently detected by secondary antibodies, which is a useful finding that would likely also be relevant for other secondary antibodies.

The authors have included the original images in the supplement, which is useful for users of the technique.

**Issues**

1. In the Results section, it should be made clear that the arrays were probed with both antibodies in the same experiment, not one antibody at a time.
2. It would be interesting how strong the signals caused by the secondary antibodies are in comparison to signals obtained in the presence of a primary antibody.
3. In comparison, the part 1 image has a much higher background than part 2. It appears that very clear signals were obtained from part 2, but not from part 1. In the part 1 image, there is considerable background and almost all positions have been slightly stained. I would recommend repeating the experiment to verify whether the weak signals obtained on part 1 can be reproduced.
4. Two secondary antibodies were used in the same experiment. Therefore, it cannot be determined which of the two antibodies gave rise to the signals on the array. This problem should be discussed.

I have read this submission. I believe that I have an appropriate level of expertise to confirm that it is of an acceptable scientific standard, however I have significant reservations, as outlined above.

# Response

**Published:** 2017-05-16

**Authors:** Gregor Kijanka

**Affiliations:** Dublin City University, Ireland

The authors would like to thank Dr. Konrad Büssow for his thorough review of this article and his helpful comments. Dr. Büssow points out that the authors should stress that both secondary antibodies were used in the same experiment using one single set of protein arrays. This experimental design issue entails that it cannot be determined which signals are caused by which antibody. We have highlighted and discussed both issues throughout the text and we performed an additional sequence analysis in an *in silico *approach to clarify the origin of the signals on the protein array. The results of these analyses are presented in the new Table 5 and Supplementary table 1 and are further discussed in the text.

Dr. Büssow has furthermore highlighted the differences in background signal between the two arrays of the protein array set. The authors have encountered similar background differences when using other sets of antibodies and serum samples and find similar discrepancies in background noise being likely due to different tissues and vectors used for the generation of the distinct expression clone libraries utilized for array 1 and 2. This issue is now specifically highlighted in the article.

# Peer review: Referee response for version 1

**DOI:** 10.5256/f1000research.8257.r12776

**Published:** 2016-03-30

**Authors:** Carsten Grötzinger

**Affiliations:** 1 Department of Gastroenterology and Molecular Cancer Research Center, Charité – Universitätsmedizin Berlin, Berlin, Germany

This article describes an experiment performed to characterize the background signals in a particular combination of three commercially available research tools: a protein macroarray on PVDF membranes used in conjunction with two antibodies used for detection. As no first antibody or serum was used in this experiment, all the signals could be attributed to unwanted, unspecific reactivity of the detection antibody combination used. A list of genes was generated from these signals that is proposed as a reference database of for other researchers.

In general, the approach is scientifically sound and feasible. Background reactivities may limit antibody-based assays and need to be accounted for. So performing a control experiment without serum or first antibody on a protein array and with just the detection antibodies makes perfectly sense to control for unspecific binding. The title of the paper is appropriate, the abstract gives enough information on the setting. The background information about the antibodies is described in enough detail. However, the narrow focus of the paper and a number of technical issues limit the quality of the paper and its utility for the readership.

**Major issues**

- The experiment was performed only once. Consequently, the reliability of the results will be limited.
- Only one specific combination of a protein macroarray with two consecutive detection antibodies was analyzed. It remains unclear, whether the results obtained would apply to other lots of the antibodies or whether they are specific for a certain preparation, limiting the benefit of this protein list as a reference database and also limiting the replication of results by other groups.
- The authors suggest that their results may also apply to other protein array systems. This claim needs substantiation, especially in the case of *E.coli* proteins derived from high-throughput cloning that do not show authentic posttranslational modification patterns and often contain extra amino acid sequences that may cause unspecific binding.
- The paper discusses cross-reactivity with human Ig genes. A sequence analysis of the other cross-reactive proteins with IgY and rabbit Ig sequences may provide evidence for the mechanisms behind this phenomenon, expanding scope and depth of this so far rather descriptive study.

**Minor issues**

- Antibody concentrations should be given explicitly, e.g. as µg/ml rather than as dilutions.
- The procedure of signal quantification and scoring needs to be described in more detail. The description states "Positive signals were localized according to the manufacturer’s protocol" - what exactly was done to identify positive signals? The pictures provided show varying background intensities as well as a number of very dark spots that do not appear in the analysis. Which algorithm was used to include or exclude signals? How were the different signal intensities attributed to the score values 1, 2 and 3?
- It would be interesting to know why this specific combination of two detection antibodies was used here: a polyclonal anti-chicken IgY antibody produced in rabbit and then a polyclonal goat anti-rabbit IgG antibody conjugated with alkaline phosphatase. Was there no conjugated anti-chicken antibody available? Every additional antibody will add to the number of unspecific reactions, so using just one instead of two may help reduce background.
- The abstract does not provide a conclusion on whether the antibodies should be used in a particular setting (see Article Guidelines For Antibody Validation Articles).

I have read this submission. I believe that I have an appropriate level of expertise to confirm that it is of an acceptable scientific standard, however I have significant reservations, as outlined above.

# Response

**Published:** 2017-05-16

**Authors:** Gregor Kijanka

**Affiliations:** Dublin City University, Ireland

The authors would like to thank Dr. Carsten Grötzinger for his very helpful observations which prompted us to perform additional *in silico* analysis resulting in an improvement of this paper. Dr Grötzinger points out that the paper has its limitations due to the fact that only one experiment has been performed leading to questions regarding reliability of data, lot-to-lot reproducibility and combinations of antibody pairs. As those issues are certainly important, however, not feasible to address in this specialized antibody validation paper, we have discussed those within the text; For instance, the lot-to-lot reproducibility of polyclonal antibodies is an important issue that needs to be taken into consideration during the experimental design of a study, it goes however, beyond the scope of this particular article. The important issue of determining the origin of the identified signals to either of the secondary antibodies tested in a single protein array experiment is now, however, addressed in more detail. We have performed an additional *in silico* analysis comparing sequence similarities between the antibody immunogens used to produce the secondary antibodies and the human proteins identified on the arrays. The analysis shed some light into the possibility that all immunoglobulin (Ig) related signals were caused by both tested secondary antibodies and others were caused by either of the two antibodies. These findings are particularly interesting, as the binding patterns of the non-labelled secondary antibody are difficult to show unless additional labelling is performed directly on the antibody. Such additional labelling might, however, impact on the antibody binding specificity. The results of those analyses, as discussed in a similar manner in the Reviewer 1 response, are presented in a new Table 5 and Supplementary Table 1 and further discussed in the text.

The authors have also addressed miner issues related to post-translational modification, antibody concentrations, signal quantification and others throughout the text.

In addition, we concluded that the antibodies should be used in a particular setting and highlighted this in the abstract as required in the Article Guidelines For Antibody Validation Articles.

# Peer review: Referee response for version 1

**DOI:** 10.5256/f1000research.8257.r12247

**Published:** 2016-02-03

**Authors:** Brigitte Hantusch

**Affiliations:** 1 Clinical Institute of Pathology, Medical University of Vienna, Vienna, Austria

This study presents data concerning the issue of secondary antibody cross-reactivity towards antigens other than desired immunoglobulins. By screening a high-throughput protein array, the authors establish the amount and identity of proteins detected by commercially available secondary antibodies, a rabbit anti-chicken antibody combined with an AP-conjugated goat anti-rabbit antibody.

**Title and Abstract**: The title might contain the information that two detection antibodies were used. The abstract represents a sound summary of the work performed.

**Article:** The methods used are described clearly, especially by showing a concise work flow as seen in table 3.

**Data:** Results are described appropriate and sufficiently. Supplementary Figures S1 and S2 have very huge size and are dispensable. The sentence about signal intensity differences due to varying protein amounts should be part of the conclusion section and also discussed more extensively.

**Conclusion**: The conclusions drawn are appropriate and concise. Briefly can some information be drawn from the kind / category of proteins falsely detected?

I have read this submission. I believe that I have an appropriate level of expertise to confirm that it is of an acceptable scientific standard.

# Response

**Published:** 2017-05-16

**Authors:** Gregor Kijanka

**Affiliations:** Dublin City University, Ireland

The authors would like to thank Dr. Brigitte Hantusch for kindly reviewing this manuscript and the helpful and detailed comments. We have discussed the signal intensity differences in more detail in the text as highlighted by Dr. Hantusch. We decided to retain the current title of the article as it points to a more general applicability of our validation approach to other antibodies.

Furthermore, we have extensively addressed Dr. Hantusch comments regarding categories of proteins detected on the protein array as part of the new *in silico* analysis as presented in Table 5 and in the supplementary Table 1
